# Efficiency and outcome of non-invasive versus invasive positive pressure ventilation therapy in respiratory failure due to chronic obstructive pulmonary disease.

**Published:** 2016

**Authors:** Valiollah Amri Maleh, Mahmood Monadi, Behzad Heidari, Parviz Amri Maleh, Ali Bijani

**Affiliations:** 1 Department of Internal Medicine, Babol University of Medical Sciences, Babol, Iran.; 2 Department of Anesthesiology, Babol University of Medical Sciences, Babol, Iran.; 3 Social Determinates of Health Research Center, Health Research Institute, Babol University of Medical Sciences, Babol, Iran.

**Keywords:** Non-invasive ventilation, Invasive mechanical ventilation, acute respiratory failure, Mortality, Chronic obstructive pulmonary disease

## Abstract

**Background:**

Application noninvasive ventilation in the patients with exacerbation of chronic obstructive pulmonary disease (COPD) reduced mortality. This case-control study was designed to compare efficiency and outcome of non-invasive (NIV) versus invasive positive pressure ventilation (IPPV) in respiratory failure due to COPD.

**Methods:**

The patients were assigned to NIV or IPPV intermittantly.The clinical parameters, including RR (respiratory rate), BP (blood pressure), HR (heart rate) and PH, PaCO2, PaO2 before and 1, 4 and 24 h after treatment were measured. Demographic information such as age, sex, severity of disease based on APACHE score, length of stay and outcome were recorded.

**Results:**

Fifty patients were enrolled in the NIV group and 50 patients in IPPV. The mean age was 70.5 in NIV and 63.9 in invasive ventilation group (p>0.05). In IPPV group, the average values of PH: PCO2: and PO2, were 7.22±0.11, 69.64 + 24.25: and 68.86±24.41 .In NIV, the respective values were 7.30±0.07, 83.94±18.95, and 60.60±19.88. In NIV group, after 1, 4 and 24 h treatment, the clinical and ventilation parameters were stable. The mean APACHE score in was IPPV, 26.46±5.45 and in NIV was 12.26±5.54 (p<0.05). The average length of hospital stay in IPPV was 15.90±10 and in NIV 8.12±6.49 days (p<0.05). The total mortality in the NIV was 4 (8%) and in IPPV, 27 patients (54%) (p<0.05).

**Conclusion:**

This study indicates that using NIPPV is a useful therapeutic mode of treatment for respiratory failure with acceptable success rate and lower mortality. The application of NIPPV reduces hospital stay, intubation and its consequent complications.

Mechanical ventilation is used as an alternative to spontaneous respiration. The main indication for initiation of mechanical ventilation is respiratory failure. According to patients' condition, either invasive or non-invasive ventilation may be used for the treatment of respiratory failure (1). Non-invasive positive pressure ventilation (NIPPV) refers to mechanical ventilation delivered through a face mask (2, 3). This modality supports ventilation without needing intubation. It is often used in patients who do not require emergency intubation (4). The main advantage of NIPPV is avoidance of intubation and therefore does not interfere with the performance of the upper airway including eating, talking and discharge of airway secretions.

Evidence collected over the past decade shows that in acute respiratory failure secondary to COPD, application of NIPPV reduces mortality and length of hospital stay. In addition incidence of ventilator-associated pneumonia, nosocomial infections such as sepsis, sinusitis decreases due to shortening of hospital stay (5-6). Efficiency of NIPPV in the treatment of respiratory failure secondary to chronic obstructive pulmonary disease (COPD) has been shown in many published studies (7-9). 

A systematic review of randomized controlled trials that compared NIPPV plus usual medical care versus medical care alone in the treatment of respiratory failure secondary to COPD exacerbation demonstrated fewer complications and shorter duration of hospital stay (10). The results of another systematic review of 14 studies in treatment of respiratory failure due to COPD exacerbations revealed that NIPPV decreases mortality, needs for intubation, rate of treatment failure, and thus provides rapid improvement of PH, PaCO2, respiratory rate and decreases duration of hospital stay (11). The available data from published studies indicate benefit of NIPPV in respiratory failure due to COPD exacerbation and so is recommended at earlier stage of respiratory failure prior to development of severe acidosis. In one study, application of NIPPV in acute respiratory failure, reduced intubation and mechanical ventilation in 20% of patients (12). 

In spite of lower complications of NIPPV compared to intensive positive pressure ventilation (IPPV), this treatment is applicable only to patients who are conscious collaborative to ventilator (4). Whereas, patients with severe respiratory failure concomitant with cardiac or respiratory arrest, loss of consciousness (GCS <10), severe upper gastrointestinal bleeding, unstable vital signs or severe cardiac arrhythmia or cases requiring emergency intubation should be considered for IPPV (4, 7, 13, 14). 

Nonetheless, intubation and mechanical ventilation is associated with several complications particularly lengthening of hospital stay with ensuing bed sores, pulmonary emboli and other untoward effects (1, 15-17). Despite the several studies in relation to the efficiency of NIPPV and IPPV for treatment of COPD exacerbations, however the data regarding the influence of NIPPV versus IPPV on the clinical and laboratory parameters of respiratory failure are scarce. For these reasons the present case- control study was performed to compare the influences of NIPPV and IPPV on clinical and laboratory parameters of respiratory failure in patients with respiratory failure admitted in ICU. 

## Methods

 The study population of this observational cohort study were recruited from September 2013 to April 2015 amongst patients with COPD who have been admitted due to respiratory failure in ICU of Ayatollah Rouhani Hospital, Babol, Iran. The study patients were allocated to either NIPPV or IPPV based on clinical conditions and arterial blood gas abnormalities. Criteria for inclusion to NIPPV were, hypoxemic respiratory failure (PaO2 < 60 and PaCO2 <45 mm HG), respiratory rate (RR) > 30 and presence of clinical symptoms indicating respiratory distress (using accessory respiratory muscles), hypercapnic respiratory failure (PH <7.3, PaCO2 > 45, RR > 30 or RR <12 per minute. Exclusion criteria were severe obesity, hemodynamic instability, presence of cardiovascular comorbidities (severe arrhythmia, myocardial infarction, unstable angina), severe respiratory failure (PH <7.2, PaO2 < 50, RR > 40 per minute, and neurologic diseases with GCS <7, cluster phobias (intolerance masks), head and neck trauma.

Patients with cardiopulmonary arrest, instability of disease conditions, inability of airways protection, airway secretions, uncooperation to NIPPV, and development of agitation during NIPPV therapy, were changed to IPPV. Data were collected regarding RR, blood pressure (BP), heart rate (HR), PH, PaCO2, PaO2 as well as demographic characteristics such as age, sex, severity of disease based on APACHE II score and concurrent respiratory diseases such as pneumonia, heart failure, pulmonary embolism and obesity hypoventilation syndrome. In the NIPPV group, ventilation was started with mask. At first, inspiratory positive airway pressure (IPAP) set on 8cm H2O and based on the respiratory rate and PaCO2, airway pressure increased gradually up to 20 cm H2O. Expiratory positive airway pressure (EPAP) set on 4cmH2O and based on SaO2 and PaO2 increased to 10 cmH2O. The purpose of changes in pressure was to maintain Sao2 levels at ranges of 90-92%. In all patients, oxygen was prescribed with a mask to attain oxygen saturation about 90%. Patients were continuously monitored with ECG, SPO2 (Peripheral O2 Saturation with pulse oximetry), RR, HR and BP and the level of consciousness. The standard treatments of the patients were continued through intravenous infusion or inhalation. 

A 5 mg dose of haloperidol was used for sedation. All mentioned parameters (RR, HR, BP, Pao2, PaCO2 and PH, APACHE II score) were assessed at baseline, first, fourth and 24 hour after initiation of treatment. The aim of this study was to determine and compare improvement of clinical and laboratory abnormalities during the first, 4th and 24th hour after initiation of treatment compared with baseline values and also to determine the duration of hospitalization and outcomes of treatment at the time of discharge in each group. In statistical analysis, chi square test was used for categorical variables and student t-test for quantitative variables.

## Results

A total of 100 consecutive patients with respiratory failure were recruited for study with respect to the inclusion criteria. Fifty patients with mean age of 63.9±13.4 years met the criteria for inclusion to NIPPV and the 50 patients with mean age of 70±13.2 years (P=0.016) who needed intubation were allocated to IPPV therapy. The number of comorbidities in IPPV group was 24 (pneumonia 12, heart failure 10, and pulmonary embolism 2) and in NIPPV group, 6 patients had CHF. Baseline clinical and laboratory features in both groups are presented in table 1. As shown in table 1, at baseline condition of patients in IPPV group were significantly worse than NIPPV regarding PH, RR, particularly APACHE II score whereas, PaCO2 was significantly higher and PaO2 significantly lower in NIPPV group. 

**Table 1:** Characteristics of demographic, clinical and laboratory measures of patients with chronic obstructive pulmonary disease treated for acute respiratory failure with non-invasive positive pressure ventilation (NIPPV) and invasive positive pressure ventilation (IPPV

**P-value**	**IPPV** **(n=50)**	**NIPPV** **(n=50)**	
0.016	70.52±13.29	63.98±13.48	Age (yr±SD)
0<0.05	62%	58%	Male (%)
0<0.5	38%	42%	Female (%)
0.001	69.64±24.25	83.94±18.95	PaCO2 (Mean±SD)
0.049	68.86±24.41	60±19.88	PaO2 (Mean±SD)
0.001	7.22±0.11	7.30±0.07	PH (Mean±SD)
0.013	28.3±8.8	24.68±5.01	Respiratory rate (Mean±SD)
0.89	98.34±20.34	92.28±14.43	Heart rate(Mean±SD)
0.014	123±40.41	138.8±18.91	Systolic BP(Mean±SD)
0.014	74.5±22.04	83.20±10.77	Diastolic BP(Mean±SD)

After initiation of treatment, most clinical and laboratory manifestations of respiratory failure responded to treatment at first hour of ventilation in both groups. However, over the first 24 hours of treatment period, percent changes in improvement of RR and HR and PH, PaCO2, PaO2 in NIPPV group was lower than IPPV. Improvement in PaCO2 and PaO2 was greater in IPPV compared with NIPPV.

Total duration of hospitalization in NIPPV was significantly lower than IPPV (8.12±6.4 vs. 15.9±10.8 days P=0.001). At endpoint, death occurred in 4 (8%) patients and treatment failure in 5 (10%) patients in NIPPV group. Whereas, death occurred in 27 (54%) patients in IPPV group (P=0.001). Mortality in both groups was associated with higher baseline APACHE II score and higher age. Overall, mean APACHE II score and age in 31 out of 100 patients (total mortalities) was significantly higher than those who survived (27.7±21.2 vs. 15.5±7.4, P=0.001) and 71.4±13.8 vs. 65.3±13.3 years, P=0.039) respectively. 

**Table 2:** Outcomes of patients treated with NIV and IPPV

	**NIV(n=50)**	**IPPV(50)**	**Pvalue**
APACHE II Score (Mean ±SD)	12.26±5.54	26.46±5.45	0.001
Predicted Mortality (%)			
Mortality n (%)	4(8%)	27(54%)	0.001
Length of stay in ICU (Mean ±SD )	8.12±6.49	15.9±10.86	0.001
Noninvasive failure n (%)	5(10%)	-	-

## Discussion

The findings of this study indicate comparable efficiency of NIPPV and IPPV in the treatment of respiratory failure in COPD. However, patients of IPPV group had more severe disease with respect to APACHE II score and so the results of treatment expected to be different. Consequently, IPPV therapy had greater potential in correcting ventilatory failure because of intubation. Nonetheless, this study showed that NIPPV was effective in the prevention of intubation and was associated with only 10% treatment failure. Therefore, the main advantage of this method of treatment is lack of intubation. Tracheal intubation increases the risk of several complications such as tracheal stenosis, upper airway injury, sinusitis, ventilator associated pneumonia, sepsis, tracheomalacia, aspiration of gastric contents (1, 5, 9, 12, 18). Currently, non-invasive ventilation is considered as an alternative method for reducing morbidity and mortality from IPPV which requires intubation. Venkatram et al. compared NIPPV and IPPV in patients with COPD exacerbations admitted to ICU (19). The two groups were matched for age, APACHE score, PH, PaCO2, PaO2, body weight, underlying disease, duration of hospitalization and mortality rates. The results demonstrated lower APACHE score and mortality rate in NIPPV versus IPPV with 5% mortality. However, 6% of patients in NIPPV group required intubation (19). 

In our study, mortality rate in IPPV was higher (54%) than Venkatram et al. which should be explained to different method of patient selection. The latter study included COPD patients with respiratory failure due to disease exacerbation whereas, in the present study patients with several underlying conditions such as ,pulmonary embolism, myocardial infarction, sepsis, cardiogenic pulmonary edema were also included. In addition, in our study the APACHE score of invasive ventilation group was greater. In both studies, patients of NIPP group had lower age, APACHE score, mortality rate, and fewer hospitalization time than invasive ventilation. Furthermore, these patients had less patients with severe acidosis but higher PCO2.

Soliman et al. studied the effectiveness of NIPPV in 27 patients with COPD. The patient s’ arterial blood gas and vital signs were monitored within 24 hours. In this study, the failure rate was 22% (6 patients). The failed patients were older and the baseline PH was lower, PCO2 and RR were higher (20).

Jason Phana et al. compared NIPPV and IPPV in patients with acute respiratory failure due to bronchiectasis. Proportion of treatment failure (changed to IPPV) in NIPPV group was 32.3%. The ratio of PaO2 to FiO2 and APACHE score were the predictors of mortality in cases with NIPPV failure (21).

Lindenaner et al. compared the outcome of NIPPV and IPPV in patients with COPD exacerbations. In this study, hospital acquired pneumonia, mortality, length of stay, cost and rate of readmission within 30 days after discharge (as an outcome) were assessed. In this study, COPD patients in NIPPV group were older and had lower risk of pneumonia, length of stay, cost, and mortality. However, readmission rates were similar in both groups. In this study, benefits of NIPPV were more evident in patients < 85 years and earlier initiation treatment (22). 

In one study of acute respiratory failure, initiation of NIPPV at the first day of disease onset was associated with success rate of 73.9% (23). In our study, the success rate was 90%. In addition, NIPPV reduced mortality rate as compared with IPPV (54% vs. 8%).However the severity of disease in IPPV was higher and so excess mortality was expected. In a study by Singh et al, the outcome predictors for non-invasive positive pressure ventilation was assessed in 50 patients with acute respiratory failure. The clinical parameters such as heart rate, respiratory rate, have been improved in 37 out of 50 patients with acute respiratory failure (74%) 24 hours after initiation of NIPPV. The remaining patients required intubation. Heart rate and respiratory rate were predictors of NIPPV treatment failure (24).

In the present study, higher age and APACHE II score were predictors of treatment failure. The results of the present study are consistent with earlier studies. However, one major limitation of studies which compared the treatment outcomes of NIPPV and IPPV is heterogeneity of patients regarding disease severity and concomitant underlying diseases, as well as different criteria applied for inclusion. Patients’ candidate for IPPV have usually more severe disease, hence, require intubation and anticipated to have greater morbidity and mortality as compared with patients who have less severe disease in NIPPV. 

However, as expected, initiation of treatment at earlier stage of disease acute respiratory failure with NIPPV reduces intubation and is expected to be associated with better outcome and lower risk of complications.

Another limitation of this study is lack of data in regard to inflammation which have major contribution in the development of morbidity and mortality. COPD is an inflammatory disease (25) and many markers of inflammation including serum C-reactive protein (26, 27) are elevated in COPD particularly those hospitalized in ICU. The results of a systematic review showed that 90% of patients at intensive care unit have elevated CRP at hospital discharge (28). High level of CRP in these patients indicates persistence of inflammation which causes ventilator limitation, muscle weakness and increased risk of comorbidities (28-30). However this issue may be applicable in both comparison groups and the two comparison groups are expected to be affected similarly and the results are less subjected to be confounded. 

In conclusion, the results of this study indicate that using NIPPV is a useful therapeutic mode of treatment for respiratory failure with acceptable success rate and lower mortality. Application of NIPPV reduces hospital stay, intubation and its consequent complications.

## Notes

**Funding: **This article is the result of a research project with code number 1314 of Babol University of Medical Sciences.
